# Measuring health systems strength and its impact: experiences from the African Health Initiative

**DOI:** 10.1186/s12913-017-2658-5

**Published:** 2017-12-21

**Authors:** Kenneth Sherr, Quinhas Fernandes, Almamy M. Kanté, Ayaga Bawah, Jeanine Condo, Wilbroad Mutale, the AHI PHIT Partnership Collaborative, Ahmed Hingora, Dominic Mboya, Amon Exavery, Kassimu Tani, Fatuma Manzi, Senga Pemba, James Phillips, Almamy Malick Kante, Kate Ramsey, Colin Baynes, John Koku Awoonor-Williams, Ayaga Bawah, Belinda Afriyie Nimako, Nicholas Kanlisi, Elizabeth F. Jackson, Mallory C. Sheff, Pearl Kyei, Patrick O. Asuming, Adriana Biney, Roma Chilengi, Helen Ayles, Moses Mwanza, Cindy Chirwa, Jeffrey Stringer, Mary Mulenga, Dennis Musatwe, Masoso Chisala, Michael Lemba, Wilbroad Mutale, Peter Drobac, Felix Cyamatare Rwabukwisi, Lisa R. Hirschhorn, Agnes Binagwaho, Neil Gupta, Fulgence Nkikabahizi, Anatole Manzi, Jeanine Condo, Didi Bertrand Farmer, Bethany Hedt-Gauthier, Kenneth Sherr, Fatima Cuembelo, Catherine Michel, Sarah Gimbel, Bradley Wagenaar, Catherine Henley, Marina Kariaganis, João Luis Manuel, Manuel Napua, Alusio Pio

**Affiliations:** 1 Department of Global Health, University of Washington, 1959 NE Pacific St, Seattle, WA USA; 2 Health Alliance International, Seattle, WA USA; 3 Ministry of Health, Maputo, Mozambique; 4 Bloomberg School of Public Health, Johns Hopkins University, Baltimore, MD USA; 5 Regional Institute for Population Studies, University of Ghana, Accra, Ghana; 6 School of Public Health, University of Rwanda, Kigali, Rwanda; 7 Department of Public Health, University of Zambia School of Medicine, Lusaka, Zambia

**Keywords:** Health system strengthening, Metrics, African Health Initiative, Ghana, Mozambique, Tanzania, Rwanda, Zambia

## Abstract

**Background:**

Health systems are essential platforms for accessible, quality health services, and population health improvements. Global health initiatives have dramatically increased health resources; however, funding to strengthen health systems has not increased commensurately, partially due to concerns about health system complexity and evidence gaps demonstrating health outcome improvements. In 2009, the African Health Initiative of the Doris Duke Charitable Foundation began supporting Population Health Implementation and Training Partnership projects in five sub-Saharan African countries (Ghana, Mozambique, Rwanda, Tanzania, and Zambia) to catalyze significant advances in strengthening health systems. This manuscript reflects on the experience of establishing an evaluation framework to measure health systems strength, and associate measures with health outcomes, as part of this Initiative.

**Methods:**

Using the World Health Organization’s health systems building block framework, the Partnerships present novel approaches to measure health systems building blocks and summarize data across and within building blocks to facilitate analytic procedures. Three Partnerships developed summary measures spanning the building blocks using principal component analysis (Ghana and Tanzania) or the balanced scorecard (Zambia). Other Partnerships developed summary measures to simplify multiple indicators within individual building blocks, including health information systems (Mozambique), and service delivery (Rwanda). At the end of the project intervention period, one to two key informants from each Partnership’s leadership team were asked to list – in rank order – the importance of the six building blocks in relation to their intervention.

**Results:**

Though there were differences across Partnerships, service delivery and information systems were reported to be the most common focus of interventions, followed by health workforce and leadership and governance. Medical products, vaccines and technologies, and health financing, were the building blocks reported to be of lower focus.

**Conclusion:**

The African Health Initiative experience furthers the science of evaluation for health systems strengthening, highlighting areas for further methodological development – including the development of valid, feasible measures sensitive to interventions in multiple contexts (particularly in leadership and governance) and describing interactions across building blocks; in developing summary statistics to facilitate testing intervention effects on health systems and associations with health status; and designing appropriate analytic models for complex, multi-level open health systems.

## Background

For over a decade, there have been calls to invest in low and middle-income country health systems to ensure stable platforms are in place to maximize evidence-based health interventions through their delivery at scale [1, 2]. Underpinning the need to strengthen fragile, resource-constrained health systems is the recognition that weak health systems impede attainment of global and national targets [3], and are insufficiently resilient to prepare for – and respond to – crises [4]. Despite this recognition and the rapid resource expansion from global health initiatives, investments in health systems have decreased relative to the overall funding envelope [5]. Stagnation in resource expansion through these initiatives raises further concerns that health system investments will only decrease, with prioritization of targeted, disease-specific efforts [6]. A lack of shared understanding of what constitutes ‘health systems strengthening,’ the potentially high cost of comprehensive health systems interventions, and a weak evidence-base linking population-level health benefits with health systems strengthening strategies undermines broad investments in this area.

To address this evidence gap and catalyze investments in health systems, the Doris Duke Charitable Foundation (DDCF) launched the African Health Initiative (AHI), which supported Population Health Implementation and Training in five diverse sub-Saharan African countries (Ghana, Mozambique, Rwanda, Tanzania and Zambia). Since 2009 (when support for Partnership implementation was initiated), these Partnerships have implemented distinct interventions designed to strengthen health systems in their respective contexts, and measured the impact of these investments on health system functioning and health outcomes [7–11].

To foster cross-site learning and produce results that may be generalizable to other low and middle-income countries (LMICs), DDCF instituted a Data Collaborative to work with the Partnerships to develop a conceptual evaluative framework, with core and common metrics mapping against this framework (including inputs/processes, outputs, outcomes and impact) to be gathered across Partnerships [12]. As part of the consultative process, Partnerships used the World Health Organization’s (WHO) health systems framework (comprised of six building blocks of service delivery; health workforce; information systems; medical products, vaccines & technologies; health financing; and leadership & governance) to identify core and common metrics on project inputs, processes and outputs [13]. Candidate core and common input, process and output metrics were reviewed and selected by the Data Collaborative and Partnerships based on 1) validity; 2) relevance and sensitivity to individual Partnership aims and processes; 3) measurement feasibility; and 4) consistency with global standards. Though Partnerships were aware of the limitations of the building blocks framework – in particular with regards to capturing interactions across blocks, and the missing element of ‘people’ – it was ultimately decided to operationalize an established framework rather than adapt or develop a framework solely for the Initiative. Note that outcome and impact indicators, as well as contextual factors, were defined by the same Data Collaborative and Partnership representatives to map against the AHI conceptual framework, though using an expanded set of reference frameworks guidance documents [12].

Despite guidelines recommending the use of standardized indicators – including measurement strategies to support monitoring and evaluation of health systems strengthening interventions [14, 15] – gaps remain in ensuring that measures are valid, sensitive to health systems interventions, and readily available at the administrative level that health systems strengthening efforts target [16, 17]. The state of knowledge on measuring health impacts and outcomes, however, is further advanced. For example, from the recent publication of 100 core indicators by the WHO – 77 of the measures fall into the impact (29) and outcome (48) categories, and only 22 are specific to health system strengthening (inputs and processes (12)), or proximally related to these inputs and processes (10) [14]. Furthermore, of the ‘health system’ indicators, 42% map against the service delivery building block; 26% health financing; 12% health information; 8% health workforce; 4% medical products, vaccines and technology; and none in leadership and governance. The lack of scientifically valid metrics reflecting health systems functioning across all of the building blocks impedes efforts to monitor and evaluate interventions designed to strengthen health systems, and build an evidence-base supporting health systems strengthening to improve health outcomes.

Attempts to evaluate health systems strengthening interventions – in terms of their effects on health systems and health status – are nascent, and lack robust, standardized methodologies for assessing complex interventions implemented at a sub-national scale [16]. Quantitative evaluations have largely focused on the impact of health systems on population health measures, including the impact of individual health system components (e.g. financing or health workforce) on health status across multiple countries [18, 19]; a partial list of health system components on health status across countries [20, 21]; or a partial list of health system components on health status over time at a national or sub-national level [22–24]. Published literature also includes qualitative endeavors assessing the perceived impact of health systems strengthening approaches broadly at a national level [25], or at the micro-level (sub-national or on individual building block components) [26]. Though important for generating evidence on the role of health systems components as determinants of population health status, current research does not adequately capture the complex, inter-connected relationships between health system building blocks, and the setting in which they are situated [27]. Realist evaluation [28] and approaches based on complexity theory [29] may have the advantage of addressing health systems interdependence and implementation context, though their ability to lead to generalizable knowledge on health systems strengthening interventions is unclear.

The purpose of this manuscript is to describe approaches from the five PHIT Partnerships to 1) measure health systems strength, and 2) demonstrate its impact on the delivery of health services and population health. Through surfacing common and distinct experiences from the Partnerships, we highlight the complexity in measuring health systems and its impact on health outcomes and impact, and discuss opportunities and priority areas for the future. By reporting on the Partnerships’ experience with operationalizing measures of health systems strengthening, and analytic approaches to link these inputs and processes with improved health services and population health, this article will be of interest to those engaged in designing and implementing complex interventions to improve the delivery of primary health care – including ministries of health, researchers, implementers, policymakers and funders.

## Methods

### Partnership approaches to strengthening health systems

By design, the five Partnerships are responsive to specific needs in their country contexts (intervention descriptions have been previously published) [7–11]. Though there may be commonalities in how Partnerships strengthen individual health system building blocks, describing differences in the health system focus of each Partnership clarifies reasons for the variations of measures of health system strengthening gathered across countries to best assess their respective interventions. At the end of the project intervention period, one to two key informants from each Partnership’s leadership team were asked to list – in rank order – the importance of the six building blocks in relation to their intervention (Table 1). Though there were differences across Partnerships, service delivery and information systems were reported to be the most common focus of interventions, followed by health workforce and leadership and governance. Medical products, vaccines and technologies, and health financing, were the building blocks reported to be of lower focus.

**Table 1:** PHIT Partnership countries’ ranking of intervention emphasis by health system building block

	Service delivery	Information systems	Health workforce	Leadership & governance	Medical products, vaccines & technologies	Health financing
Ghana	1	4	5	3	6	2
Mozambique	3	2	5	1	6	4
Rwanda	1	2	3	4	5	6
Tanzania	2	5	1	3	4	6
Zambia	3	2	1	4	5	6
Median	2	2	3	3	5	6

To understand differences in data availability and situate selected measures of health system strength and its impact within each project, the same key informants were requested to list – in rank order – the administrative level of the health system prioritized for Partnership interventions (Table 2). Notably, two Partnerships (Ghana and Tanzania) emphasized the community level in their intervention design; another two (Rwanda and Zambia) emphasized the health facility level; and one (Mozambique) emphasized the district level. Only one Partnership intervened at the provincial (Mozambique) and national (Rwanda) levels.

**Table 2:** PHIT Partnership countries’ ranking of administrative unit of emphasis by health systems building block

	Community	Health facility	District & sub-district	Province	National
Ghana	1	2	3	0	0
Mozambique	0	2	1	3	0
Rwanda	3	1	2	NA	4
Tanzania	1	3	2	0	0
Zambia	2	1	3	0	0
Median	1.5	2	2	3	4

*NA* not applicable, 0 = not included in Partnership design, and not included in calculation of median

## Results

### Partnership measures and data collection approaches

Because all Partnerships were asked to include under-five mortality (5q0) as the primary study outcome, and all agreed on core outcome and impact measures early in the Initiative, outcome and impact measures varied little across countries. However, given the diverse implementation settings and intervention designs, there are notable differences in Partnership measures of health system strength (Table 3) and data collection approaches (Table 4). For three building blocks (health financing; medical products, vaccines and technologies; and service delivery), there were commonalities in measures across countries, which is likely due to both more established measures that are feasible to routinely collect, as well as agreement on core metrics in these areas early in the Initiative. However, there were differences in Partnership measures across the remaining building blocks of leadership and governance, health workforce, and health information systems, reflecting the lack of established metrics (e.g. leadership and governance), differing levels of importance given Partnership interventions (e.g. health information), and different approaches of Partnerships themselves (e.g. health workforce).

**Table 3:** PHIT Partnership countries’ health systems strengthening measures by health systems building block^a^

		Ghana	Mozambique	Rwanda	Tanzania	Zambia
Service Delivery	Indicators	• Service utilization & quality for selected programs • Emergency referrals • Cause of maternal deaths	• Service utilization for selected programs • Patient satisfaction with PHC services • Average wait/consult time for PHC services	• Service utilization for selected programs • Facility capacity • Quality of care: Appropriate diagnosis and treatment	• Service utilization for selected programs • Time use of CHWs • Quality of IMCI	• Service utilization for selected programs • Patient satisfaction with PHC services • Quality of care: readiness and quality audit
	Sources	-HMIS -Project information system -Mortality audits -Community surveys	-Facility surveys -HMIS -Community surveys	-Facility surveys -HMIS -Community surveys	-Health & Demographic Surveillance System -Facility surveys -Project information system -Community surveys	-Facility surveys -Community surveys
Information Systems	Indicators	*Not measured*	• Facility & district data quality	• Community & facility data quality	• % monthly CHW reports submitted	• Timely health facility and CHW reporting
	Sources		-Data quality audits	-Data quality audits	- Project information system	-Project information system
Health Workforce	Indicators	• CHW coverage (stratified by community health nurse and midwife) • # and % of CHWs trained in IMCI & supervision frequency	• Health worker per capita by cadre • Frequency of supervision visits by district	• Health worker presence • Retention • Competency, knowledge & skills	• CHW presence during supervision • CHWs selected/trained/training results/retained • Supervisors trained & working • Frequency of supervision visit to CHWs	• Health worker motivation • Health workers trained in previous 12 months
	Sources	-Community survey -Project information system	-Project information system	-Facility surveys -Project information system	-Project information system	-Facility surveys
Leadership & Governance	Indicators	• Perceptions of governance and leadership changes	• Data utilization • % of district management teams fully staffed • Turnover of district and provincial management teams	• Data utilization • Appropriate use of resources	*Not measured*	• Evidence-based planning • Level of corruption
	Sources	-Process evaluation	-Special study -Project information system	-Special study -Policy analysis		-Facility surveys
Medical Products, Vaccines & Technologies	Indicators	• Continuous stocks of essential commodities (tracer medicines and equipment)	• Continuous stocks of essential commodities (tracer medicines and equipment)	• Continuous stocks of essential commodities (tracer medicines and equipment)	• Continuous stocks of essential commodities (tracer medicines and equipment)	• Continuous stocks of essential commodities (tracer medicines and equipment)
	Sources	-Project information system -Community survey -Facility surveys	-Facility surveys	-Facility surveys	-CHW stock card review	-Facility surveys
Health Financing	Indicators	• Total costs in intervention area • Incremental project cost	• Total costs in intervention area • Incremental project cost	• Total costs in intervention area • Cost by source • Incremental project cost	• Total costs in intervention area • Incremental project cost	• Total costs in intervention area • Incremental project cost
	Sources	-Project information system	-Ministry of Health reports -Project information system	-Ministry of Health & local government reports -Project information system	-Project information system	-Facility surveys

^a^Further details on the following metrics are available as a web annex to the following publication(Bryce et al., [12]): Medical products, vaccines & technologies; *CHW* Community Health Worker, *HMIS* Health Management Information System, *PHC* Primary Health Care, *IMCI* Integrated Management of Childhood Illnesses

**Table 4:** Data collection strategy by PHIT Partnership country: Frequency and Sampling

		Ghana	Mozambique	Rwanda	Tanzania	Zambia
Facility-based data collection	Frequency	• CHW time-motion survey: Baseline • Health facility survey: Baseline and midline	• Patient exit and time-motion survey: Baseline and endline • Health facility survey: Annual service provision assessment and data quality audit	• Health facility survey: Quarterly	• CHW time-motion survey: Baseline • Health facility survey: Baseline and midline	• Health facility survey: Baseline, midline, and endline
	Sampling	Time-motion survey: Intervention areas Facility survey: Intervention and comparison areas	Patient exit & time-motion survey: Intervention and comparison areas Facility survey: 27 purposively selected facilities, intervention area	All 22 facilities in intervention area	Time-motion survey: Intervention areas Facility survey: Sample of 107 (baseline) and 141 (midline) facilities in intervention & comparison areas	All intervention and comparison areas^a^
Special studies	Frequency	Qualitative process evaluation	Qualitative process evaluation: endline	Qualitative process evaluation Policy analysis	Qualitative process evaluation	Qualitative process evaluation
	Sampling	Key informant interviews & focus group discussions with district and national health leaders	Key informant interviews with purposively selected stakeholders in intervention area	Key informant interviews with purposively selected local and national health leaders	Key informant interviews & focus group discussions with community, facility and district health leaders	Key informant interviews with facility and district leaders
Community Surveys / Demographic Surveillance System	Frequency	Community survey: Baseline & endline Demographic Surveillance system (Navrongo): Semi-annual	Community survey: Baseline, midline (2 surveys), & endline	Community survey: Baseline & endline	Health and Demographic Surveillance System: Semi-annual	Community survey: Approximately annual
	Sampling	Intervention and comparison areas	Intervention and comparison areas	Intervention and comparison areas	Intervention and comparison areas	Intervention and comparison areas
Health Management Information System	Frequency	Monthly facility reports	Monthly facility reports	Monthly facility reports	Monthly CHW activity reports	Monthly facility reports
	Sampling	Full capture for all intervention and control facilities	Full capture for all facilities in intervention area	Full capture for all facilities in intervention area	Full capture for all CHWs in intervention area	Full capture for all facilities in intervention and comparison areas
Project Monitoring System	Frequency	Ongoing training, supervision, program and financial reports Ongoing contextual data tracking	Ongoing training, supervision program and financial reports Ongoing contextual data tracking	Ongoing training, supervision program and financial reports Ongoing contextual data tracking	Ongoing training, supervision program and financial reports Ongoing contextual data tracking	Ongoing training, supervision program and financial reports Ongoing contextual data tracking
	Sampling	Routine activity reports: Intervention area Contextual data: Intervention and comparison areas	Routine activity reports: Intervention area Contextual data: Intervention and comparison areas	Routine activity reports: Intervention area Contextual data: Intervention and comparison areas	Routine activity reports: Intervention area Contextual data: Intervention and comparison areas	Routine activity reports: Intervention and comparison areas Contextual data: Intervention and comparison areas

^a^Note: As Zambia used a stepped-wedge design, intervention and comparison facilities vary over time

All countries relied on a mix of sources for health systems data, including facility surveys, population-based surveys, internal monitoring systems, and government health management information systems (HMIS). Similarities across Partnerships included a reliance on facility surveys to populate service readiness data (including quality of care and patient satisfaction), and continuous stock of essential supplies and commodities. The frequency of facility assessments, sampling approach, and inclusion of comparison areas differed by country context, and because of the stepped wedge design, only Zambia carried out facility surveys in all intervention and comparison areas. All Partnerships noted the value of facility assessments as a principal source of health systems data; however, respondents voiced concern about the validity of the findings from facility assessments, as well as the large quantity of data that are not readily summarized for further analysis. The use of routine data was found to be efficient, and leveraged health information system improvement activities.

All countries relied on population-based surveys for outcomes and impact data, and to estimate service utilization for programs most relevant to their theory of change. Two partnerships relied on existing population-based surveys as their primary data source (e.g. Demographic and Health Surveys), which gained efficiencies, and in the case of Rwanda, was bolstered through over-sampling. Reliance on national surveys did pose challenges, however, in terms of 1) having limited flexibility in modules included in the surveys, 2) the relatively limited power of national community surveys considering sub-national (and at times sub-provincial) intervention and comparison areas, and 3) having no control over the timing of surveys. As a result, certain measures (e.g. having four or more antenatal care visits during the last pregnancy, which was not included in a Multi-Indicator Cluster Survey in Mozambique) could not be included as core to the Initiative. Mozambique is also implementing a costly endline population-based survey, as there is no national survey that includes core initiative metrics timed with the end of the intervention. Three countries (Ghana, Mozambique and Tanzania) carried out time-motion studies to quantify human resource use patterns, including wait and consult times. Partnerships highlighted the resource requirements to collect these data, and though useful in understanding staffing patterns, there were questions about the sensitivity of time-motion measures to program interventions in settings with severe personnel shortages. There were additional concerns about the consistently high level of reported patient satisfaction, which is of limited use for informing targeted action or for Partnership evaluations.

### Summarizing health systems strength

Given the complexity of health systems, approaches to measure health system strength must be multi-faceted and include multiple indicators across the six building blocks, which presents a challenge in succinctly summarizing health system strength. There are two principal needs for summary measures reflecting health systems strength, including that 1) they enable rapid monitoring of health system capacity for targeted action by ministries of health, and 2) reducing the hundreds of health systems indicators into a limited set of metrics is required to quantify both the effect of interventions on health systems strength, and between health systems strength and measures of health service delivery and population health.

**Fig. 1:**
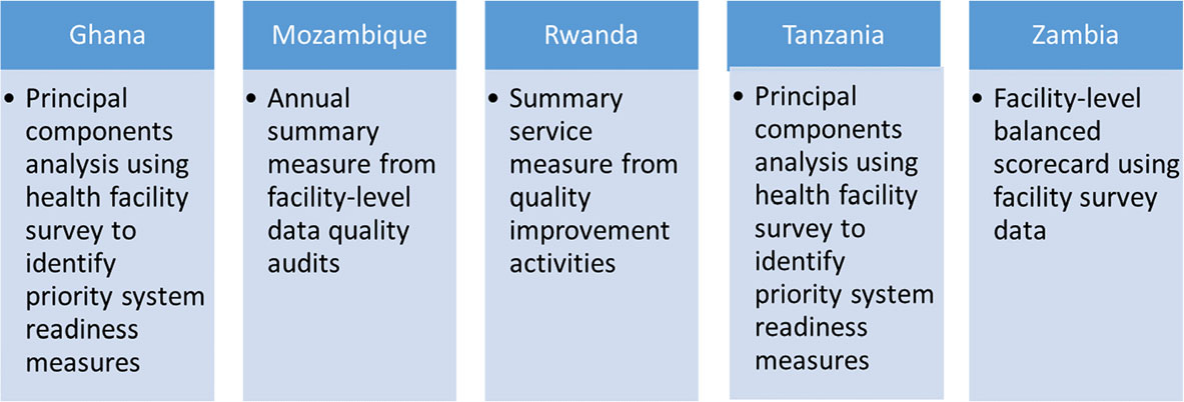
Novel summary measures of health systems strength by PHIT Partnership

The five Partnerships all employed techniques to summarize health systems data, though approaches differed across countries (see Fig. 1). Using principal components analysis on national health facility surveys, two Partnerships (Ghana and Tanzania) constructed composite indices that aimed to provide robust measures of health system capabilities [30, 31]. A limitation of these data, however, is that the surveys were not carried out to the dispensary level (the level of focus in both Partnerships). A third Partnership (Zambia) adapted the WHO balanced scorecard [15] to summarize data from health facility surveys (implemented as part of the Partnership’s evaluation plan) into 19 measures that crossed seven health system domains [32, 33]. Efforts were made to summarize data within building blocks. In Mozambique, where improving data quality was a priority, a summary measure was developed that collapsed the dimensions of data availability and concordance using four indicators from facility reports over 12 months into one facility-level proportion [34, 35]. In Rwanda, the Partnership developed a composite measure of service quality as part of their quality improvement approach (specifically to target facility-level improvement efforts). The Rwanda Partnership also developed a micro-level composite indicator for neonatal health screening that summarized performance at the facility level for further targeted action. Only one measure collected across all countries cut across all building blocks – the total cost of health services, and the incremental contribution of each PHIT Partnership.

### Approaches to associate health system strengthening with outcome and impact measures

It is beyond the scope of this article to describe the Partnerships’ analysis plans. However, given the experience of the Partnerships in designing analytic approaches to assess the effect of their complex interventions, the following section describes novel approaches used to incorporate measures of health systems strength into Partnership analytic plans. All Partnerships planned to assess 1) if the intervention is associated with improvements in population-level health status; 2) if health systems were strengthened over time in intervention areas compared with comparison areas; and 3) if health systems strengthening is associated with improvements in health service coverage and population-level health status (5q0 in all countries, though countries will also assess neonatal (NN) and infant (1q0) mortality – and in the case of Zambia and Tanzania – adult mortality). All Partnerships assessed improvements in collected measures of health systems strength by building block, though Ghana, Tanzania and Zambia planned to operationalize their summary measure of health systems strength in their analyses.

At the time of writing, Partnerships are still collecting final outcome data, or are carrying out final analyses, though initial work has generated insights into what has worked well in evaluating complex health systems interventions in the five countries, as well as challenges in this area of inquiry. Partnerships noted that a prospective, mixed methods approach is essential to understand if health systems are improving, and to unpack the middle of ‘how’ and ‘why’ interventions are or are not leading to improvements in service delivery coverage and health status. There were noted challenges, including questions on whether and how to adjust for contextual factors – including the presence of other initiatives – to enable attribution of effect to Partnership interventions. Second, Partnerships noted difficulties in teasing apart the relative contributions of different components of the health system on overall impact, given the interdependence across building blocks, and that the relative contribution of different components is likely unequal (with ‘dose’ varying by Partnership design, and over time). Inherent in this challenge is recognition that critical attributes of a health system – such as trust, resilience, quality, and leadership – are not easily quantified, and as critical for routine functioning across other building blocks, likely confound the assessment of intervention effects on individual building blocks. In addition, given the complexity of Partnership interventions, there were differences of opinions about using adaptive designs allowing for innovations based on lessons learned during implementation, versus strict adherence to the initial program design. There are no easy answers to these questions, though we expect some clarity as the field of evaluation sciences develops.

## Discussion

Here we present how five Partnerships supported through the Doris Duke Charitable Foundation’s African Heath Initiative approached the collection of a set of core and common metrics for health systems strengthening, and approaches to simplify and operationalize these measures to assess the effect of interventions on health system functioning and population health. Each Partnership was unique in intervention design and setting (located in five sub-Saharan African countries), but shared a list of core (shared by all Partnerships) and common (shared by multiple Partnerships) metrics that provides a solid set of experiences to learn about measuring health systems strength and its broader impact. The shared experience of the Partnerships demonstrates the difficulties in quantifying health system inputs and processes, and health system strength, due to a lack of scientifically valid measures that are sensitive to varied, complex interventions in multiple health system contexts. While some building blocks (e.g. economic inputs), outcomes and health status have established measures and data sources, others (e.g. governance and information systems) are particularly challenging. The PHIT Partnership experience provides examples of health system measures – identifying advancements in some areas, and needs for further development – and describes novel approaches to summarize health system measures for operationalization in evaluation of complex interventions. Ultimately, assessing health systems and their impact requires mixed-methods, relying on data from multiple, complementary sources.

Early in the Initiative, Partnerships agreed to use the WHO health system framework to orient the selection of core and common indicators. At the end of this Initiative, Partnerships reflected on the strengths and weaknesses of this framework. The building blocks framework was found to be useful in separating out the ingredients of health systems, and identifying key measures for these domains to enable Partnerships to document inputs and processes associated with their interventions, and quantifying the impact of these inputs and processes with outcomes and impact. However, limitations with the building blocks framework as a guide for metrics for health systems strengthening were identified. Partnerships noted that – though the framework isolated key ingredients in the health system – it did not capture the interaction between building blocks. Feedback loops (both positive and negative) between building blocks are important in the context of measuring health systems strength, given the likely interactions in intervention effects across blocks and the inability to impact building blocks in isolation (for example, improve health workforce without also improving facility conditions and/or leadership). Furthermore, the framework was found to inadequately capture implementation context – including social and organizational context. As a predominately supply-side model, the framework does not adequately capture the block of ‘people’, including community linkages, linkages with non-formal leaders, the role of the private sector, and the importance of demand creation in bridging health needs with service availability. Measuring health systems strength requires a better understanding of how health systems support community needs, and how communities contribute to health systems strengthening [2]. Despite its limitations, the building block framework was useful to guide the complex process of identifying core and common measures of health systems strength, and could be improved upon by adapting the framework to be an open system that recognizes linkages between its components, and with the broader context in which it is situated. Alternative evaluation approaches – such as realist evaluation [28] and evaluations built on complexity science [29] – are also relevant in explicitly addressing systems complexity and context.

The experience of the five Partnerships enriches previous efforts to develop metrics for health systems strengthening by demonstrating different collection strategies, and highlighting measures that are feasible, valid and sensitive to interventions across multiple settings. Current global indicator standards [14, 36] are weighted towards outcomes and health status – and within the building blocks – towards financing input levels and health workforce numbers and their distribution, which reflects the greater availability and validity of these indicators relative to those across the other health system building blocks. There was consistency across Partnerships in terms of measures of health information, medical technologies and service delivery, collected via facility surveys that are expensive, inconsistently conducted, and in the examples presented here were not representative in multiple countries (either not reflective of the level of Partnership intervention, or did not include both intervention and comparison areas). The lack of scientifically valid and appropriate measures for the building block of ‘governance and leadership,’ (including indicators related to leadership and management at sub-national levels, beyond the existence of up-to-date national policies, that can be operationalized for analysis), has been noted elsewhere [37, 38]. This gap is especially worrisome given that leadership and governance is critical to strong, effective health systems, and likely reverberates across all other building blocks [39]. It is a priority to develop and validate across multiple contexts new measures for leadership and governance, including how different types of evidence are used by decision makers.

All country teams developed approaches to summarize data across health system building blocks or within individual blocks (aligning with Partnership theories of change), and plan to incorporate these summary measures in final analysis. These examples provide guidance for others researching health systems, and working to operationalize summary systems measures in analytic procedures. Further work is needed to validate these summary measures of health systems strength, which is a still-forming methodologic frontier that must address metric performance given varied contexts and health system complexity [16]. A common methodologic challenge across teams related to the primary outcome measure – 5q0 – especially in countries without a health and demographic surveillance system, where national-level community surveys do not capture these relatively rare events with sufficient precision, at meaningful time intervals, and at the district level (where Partnerships intervene). As has been described elsewhere, 5q0 may be a sub-optimal measure to evaluate complex health system interventions, as secular trends in 5q0 may hinder detection of reductions due to the interventions. Furthermore, multiple pathways to impact 5q0, and concurrent health and non-health sector inputs, may hinder attribution to specific interventions [40].

Ultimately, health systems are a means to an end – as delivery platforms to ensure equitable access to high quality, evidence-based health care, with the end goal of improving the health of populations. However, investments in health systems continue to be seen as overly-complex ‘black boxes’ without clear evidence on what works, and ‘black holes’ requiring substantial resource inputs (potentially at the expense of other priorities) [5, 41]. The dearth of evidence on how complex interventions improve health system functioning, and ultimately save people lives, reinforces this perception [2, 27], underscoring the need to establish a core set of validated health systems indicators across building blocks, and analytic approaches that explore interaction across the building blocks and with the outer context [42]. Validated measures and appropriate analytic techniques are essential to continue to build a body of evidence on how to strengthen health systems, and the potential benefits on improved health service coverage and population health impact; to establish targets for health system strengthening; and ensure that substantial resource investments through global health initiatives and national budget allocations are maximized.

## Conclusions

The African Health Initiative was launched to meaningfully strengthen health systems, and to generate evidence on effective approaches to develop health systems that lead to measurable improvements in health status. Implicit in this objective is the ability to measure stronger health systems, and associate these measures with population-level health outcomes. Measuring health system strengthening is complex, and while the WHO framework is useful, it is not sufficient to describe how the parts function as a system. Innovative approaches to develop health systems indicators, validated against health outcomes, are vital, and with the final results of the African Health Initiative, some of these indicators will be available.

## Abbreviations

AHI: African Health Initiative
DDCF: Doris Duke Charitable Foundation
HMIS: Health management information system
LMICs: Low- and middle income- countries
NN: Neonatal
PHIT: Population Health Implementation and Training Partnerships
WHO: World Health Organization

## References

[1] Travis P, Bennett S, Haines A, Pang T, Bhutta Z, Hyder AA, Pielemeier NR, Mills A, Evans T (2004). Overcoming health-systems constraints to achieve the millennium development goals. Lancet.

[2] De Savigny D, Adam T (2009). Systems thinking for health systems strengthening.

[3] Atun R, Weil DE, Eang MT, Mwakyusa D (2010). Health-system strengthening and tuberculosis control. Lancet.

[4] Kruk ME, Myers M, Varpilah ST, Dahn BT (2015). What is a resilient health system? Lessons from Ebola. Lancet.

[5] Hafner T, Shiffman J (2013). The emergence of global attention to health systems strengthening. Health Policy Plan.

[6] Institute For Health Metrics And Evaluation, IHME (2014). Financing global health 2013: transition in an age of austerity.

[7] Awoonor-Williams JK, Bawah AA, Nyonator FK, Asuru R, Oduro A, Ofosu A, Phillips JF (2013). The Ghana essential health interventions program: a plausibility trial of the impact of health systems strengthening on maternal & child survival. BMC Health Serv Res.

[8] Sherr K, Cuembelo F, Michel C, Gimbel S, Micek M, Kariaganis M, Pio A, Manuel JL, Pfeiffer J, Gloyd S (2013). Strengthening integrated primary health care in Sofala, Mozambique. BMC Health Serv Res.

[9] Drobac PC, Basinga P, Condo J, Farmer PE, Finnegan KE, Hamon JK, Amoroso C, Hirschhorn LR, Kakoma JB, Lu C, Murangwa Y, Murray M, Ngabo F, Rich M, Thomson D, Binagwaho A (2013). Comprehensive and integrated district health systems strengthening: the Rwanda population health implementation and training (PHIT) partnership. BMC Health Serv Res.

[10] Ramsey K, Hingora A, Kante M, Jackson E, Exavery A, Pemba S, Manzi F, Baynes C, Helleringer S, Phillips JF (2013). The Tanzania connect project: a cluster-randomized trial of the child survival impact of adding paid community health workers to an existing facility-focused health system. BMC Health Serv Res.

[11] Stringer JS, Chisembele-Taylor A, Chibwesha CJ, Chi HF, Ayles H, Manda H, Mazimba W, Schuttner L, Sindano N, Williams FB, Chintu N, Chilengi R (2013). Protocol-driven primary care and community linkages to improve population health in rural Zambia: the better health outcomes through mentoring and assessment (BHOMA) project. BMC Health Serv Res.

[12] Bryce J, Requejo JH, Moulton LH, Ram M, Black RE, Population Health, I. & Training - Africa Health Initiative Data, C (2013). A common evaluation framework for the African health initiative. BMC Health Serv Res.

[13] World Health Organization (2007). Everybody's business: strengthening health systems to improve health outcomes: WHO’s framework for action.

[14] World Health Organization (2015). Global reference list of 100 Core health indicators.

[15] World Health Organization (2010). Monitoring the building blocks of the health system: a handbook of indicators and their measurement strategies.

[16] Samb B, Evans T, Dybul M, Atun R, Moatti JP, Nishtar S, Wright A, Celletti F, Hsu J, Kim JY, Brugha R, Russell A, Etienne C, World Health Organization Maximizing Positive Synergies Collaborative, G (2009). An assessment of interactions between global health initiatives and country health systems. Lancet.

[17] Kruk ME, Freedman LP (2008). Assessing health system performance in developing countries: a review of the literature. Health Policy.

[18] Stuckler D, Basu S, Mckee M (2010). Drivers of inequality in millennium development goal progress: a statistical analysis. PLoS Med.

[19] Anand S, Barnighausen T (2004). Human resources and health outcomes: cross-country econometric study. Lancet.

[20] Muldoon KA, Galway LP, Nakajima M, Kanters S, Hogg RS, Bendavid E, Mills EJ (2011). Health system determinants of infant, child and maternal mortality: a cross-sectional study of UN member countries. Glob Health.

[21] Wang L (2003). Determinants of child mortality in LDCs: empirical findings from demographic and health surveys. Health Policy.

[22] Fernandes QF, Wagenaar BH, Anselmi L, Pfeiffer J, Gloyd S, Sherr K (2014). Effects of health-system strengthening on under-5, infant, and neonatal mortality: 11-year provincial-level time-series analyses in Mozambique. Lancet Glob Health.

[23] Masanja H, De Savigny D, Smithson P, Schellenberg J, John T, Mbuya C, Upunda G, Boerma T, Victora C, Smith T, Mshinda H (2008). Child survival gains in Tanzania: analysis of data from demographic and health surveys. Lancet.

[24] Kumalija CJ, Perera S, Masanja H, Rubona J, Ipuge Y, Mboera L, Hosseinpoor AR, Boerma T (2015). Regional differences in intervention coverage and health system strength in Tanzania. PLoS One.

[25] Craveiro I, Dussault G (2016). The impact of global health initiatives on the health system in Angola. Glob Public Health.

[26] Scott V, Schaay N, Olckers P, Nqana N, Lehmann U, Gilson L (2014). Exploring the nature of governance at the level of implementation for health system strengthening: the DIALHS experience. Health Policy Plan.

[27] Adam T, Hsu J, De Savigny D, Lavis JN, Rottingen JA, Bennett S (2012). Evaluating health systems strengthening interventions in low-income and middle-income countries: are we asking the right questions?. Health Policy Plan.

[28] Pawson R, Tilley N (2001). Realistic evaluation bloodlines. Am J Eval.

[29] Walton M (2016). Expert views on applying complexity theory in evaluation: opportunities and barriers. Evaluation.

[30] Boyer C, Jackson E, Bawah A, Schmitt M, Awoonor-Williams J, Phillips J. Estimating indices of health system readiness: an example from rural northern Ghana. Lancet Glob Health. 3:S14.

[31] Jackson EF, Siddiqui A, Gutierrez H, Kante AM, Austin J, Phillips JF (2015). Estimation of indices of health service readiness with a principal component analysis of the Tanzania service provision assessment survey. BMC Health Serv Res.

[32] Mutale W, Godfrey-Fausset P, Mwanamwenge MT, Kasese N, Chintu N, Balabanova D, Spicer N, Ayles H (2013). Measuring health system strengthening: application of the balanced scorecard approach to rank the baseline performance of three rural districts in Zambia. PLoS One.

[33] Mutale W, Stringer J, Chintu N, Chilengi R, Mwanamwenge MT, Kasese N, Balabanova D, Spicer N, Lewis J, Ayles H (2014). Application of balanced scorecard in the evaluation of a complex health system intervention: 12 months post intervention findings from the BHOMA intervention: a cluster randomised trial in Zambia. PLoS One.

[34] Gimbel S, Micek M, Lambdin B, Lara J, Karagianis M, Cuembelo F, Gloyd SS, Pfeiffer J, Sherr K (2011). An assessment of routine primary care health information system data quality in Sofala Province, Mozambique. Popul Health Metr.

[35] Wagenaar BH, Gimbel S, Hoek R, Pfeiffer J, Michel C, Manuel JL, Cuembelo F, Quembo T, Afonso P, Porthe V, Gloyd S, Sherr K (2015). Effects of a health information system data quality intervention on concordance in Mozambique: time-series analyses from 2009-2012. Popul Health Metr.

[36] United Nations Department Of Economic And Social Affairs. 2015. IAEG-SDG Open Consultation on Green Indicators [Online]. Available: http://unstats.un.org/sdgs/iaeg-sdgs/open-consultation-2 [Accessed 28 Jan 2016].

[37] Phillips JF, Sheff M, Boyer CB (2015). The astronomy of Africa's health systems literature during the MDG era: where are the systems clusters?. Glob Health Sci Pract.

[38] Alliance For Health Policy And Systems Research (2008). Neglected health systems research: governance and accountability.

[39] Balabanova D, Mills A, Conteh L, Akkazieva B, Banteyerga H, Dash U, Gilson L, Harmer A, Ibraimova A, Islam Z, Kidanu A, Koehlmoos TP, Limwattananon S, Muraleedharan VR, Murzalieva G, Palafox B, Panichkriangkrai W, Patcharanarumol W, Penn-Kekana L, Powell-Jackson T, Tangcharoensathien V, Mckee M (2013). Good health at low cost 25 years on: lessons for the future of health systems strengthening. Lancet.

[40] English M, Irimu G, Wamae A, Were F, Wasunna A, Fegan G, Peshu N (2008). Health systems research in a low-income country: easier said than done. Arch Dis Child.

[41] Frenk J (2010). The global health system: strengthening national health systems as the next step for global progress. PLoS Med.

[42] Gilson L, Hanson K, Sheikh K, Agyepong IA, Ssengooba F, Bennett S (2011). Building the field of health policy and systems research: social science matters. PLoS Med.

